# Italian Physicians’ Perceptions about the Role of Asciminib in Later Lines Chronic Myeloid Leukemia in Clinical Practice: A GIMEMA Survey

**DOI:** 10.3390/jcm12165267

**Published:** 2023-08-13

**Authors:** Massimo Breccia, Alfonso Piciocchi, Elisabetta Abruzzese, Daniela Cilloni, Monica Messina, Stefano Soddu, Fausto Castagnetti, Fabio Stagno, Paola Fazi, Alessandra Iurlo, Giovanni Caocci, Antonella Gozzini, Tamara Intermesoli, Mariella D’Adda, Fabrizio Pane

**Affiliations:** 1 Department of Translational and Precision Medicine, Sapienza University, 00161 Rome, Italy; 2 GIMEMA Foundation, 00182 Rome, Italy; a.piciocchi@gimema.it (A.P.); m.messina@gimema.it (M.M.); s.soddu@gimema.it (S.S.); p.fazi@gimema.it (P.F.); 3 Unità Operativa Complessa U.O.C. Ematologia, Ospedale S. Eugenio, 00144 Rome, Italy; elisabetta.abruzzese@uniroma2.it; 4 Ematologia, Azienda Ospedaliera Mauriziano Umberto I, 10128 Turin, Italy; daniela.cilloni@unito.it; 5 Hematology Unit, IRCCS Azienda Ospedaliero, University of Bologna, 40138 Bologna, Italy; fausto.castagnetti@unibo.it; 6 Department of Medical and Surgical Sciences, University of Bologna, 40138 Bologna, Italy; 7 CTC U.O di Ematologia con Trapianto di Midollo Osseo, 95123 Catania, Italy; fsematol@tiscali.it; 8 Ematologia, Fondazione IRCCS CA’ Granda, Ospedale Maggiore Policlinico, 20122 Milano, Italy; alessandra.iurlo@policlinico.mi.it; 9 Azienda Ospedaliera Brotzu, Presidio Ospedaliero A. Businco, Struttura Complessa Ematologia E CTMO, 09124 Cagliari, Italy; giovanni.caocci@unica.it; 10 Struttura Ospedaliera Dipartimentale Ematologia, AOU Careggi, 50139 Firenze, Italy; antonella.gozzini@unifi.it; 11 Struttura Complessa Ematologia, Ospedale di Bergamo, ASST Papa Giovanni XXIII, 24127 Bergamo, Italy; tintermesoli@asst-pg23.it; 12 UO Ematologia, ASST Degli Spedali Civili di Brescia, 25123 Brescia, Italy; marielladadda@libero.it; 13 Unità Operativa Complessa Ematologia, AOU Federico II, 80131 Napoli, Italy; fabrizio.pane@unina.it

**Keywords:** chronic myeloid leukemia, later lines, asciminib

## Abstract

Unmet needs remain in later lines chronic myeloid leukemia (CML): the response rate and the overall survival of resistant patients in the chronic phase who changed a second-generation TKI in the second line with another TKI with similar action are usually poor, while the off-target toxicities and the potential development of mutations increase. The recent approval of asciminib, a STAMP inhibitor, in the third line, has the potential to soon change the therapeutic algorithm for this subset of patients. Here, we report the results of a GIMEMA survey assessing the number of patients currently treated in the third line in Italy, the current approach in later lines by Italian physicians, and the future role of this drug according to the reason to switch to asciminib (resistance and/or intolerance), as well as the perceptions about the future position of this agent.

## 1. Introduction

Tyrosine kinase inhibitors (TKIs) ensure to the majority of chronic myeloid leukemia (CML) patients a life expectancy superimposable to the normal population [1]. However, a significant proportion of patients experience failure to treatment with first- or second-generation TKIs [2]. The switch from a second line with a second-generation TKI (2gen TKI) to another 2gen TKI is usually associated with low response rates and reduced overall survival (OS) [3]. Unmet needs remain for later lines CML: all the available TKIs have possible off-target effects in the long-term, and the sequential TKI treatment may allow the emergence of mutant clones, such as the T315I mutation reported with a frequency of 3–15% [4,5]. These unmet needs motivate the search for additional drugs to manage this subset of patients. Asciminib, the first example of an allosteric inhibitor, has been recently approved by the FDA and EMA as third-line treatment in chronic-phase CML (CP-CML) patients after resistance and/or intolerance to two previous lines of treatment [6]. Recently, a median 4-year follow-up of a phase 1a trial has been reported for 115 non-T315I mutated patients. About 70% of the patients remained on treatment, and in patients without a baseline response, 61.3% of them achieved a *BCR::ABL1* ratio <1%, 61.6% a major molecular response (MMR), and 33.7% a deep molecular response. The long-term data also showed a manageable drug: the most common side effects recorded were increased pancreatic enzymes (22.6%), thrombocytopenia (13.9%), and hypertension (13%), but only 11% of the patients discontinued it due to the onset of adverse events [7]. The phase 3 ASCEMBL study at a 96-week follow-up comparing asciminib and bosutinib showed an MMR rate superior in the asciminib arm together with a favorable safety profile. The MMR rate at 96 weeks of follow-up was 37.6% vs. 15.8% of bosutinib with molecular responses deepened over time regardless of previous lines of treatment received [8]. The toxicity profile reported showed only thrombocytopenia and neutropenia as the main adverse events. The event-free survival (EFS) reported at 2 years was 57.4% for patients treated with asciminib vs. 25.2% for bosutinib. A recent sub-analysis of the trial showed the dynamics of response. Of 60 patients who entered the trial with a *BCR::ABL1* ratio <10%, 18 (30%), 24 (40%), and 36 (60%) reached an MMR at weeks 12, 24, and 96, respectively. Of 97 patients with a *BCR::ABL1* ratio >10%, 10 (10.3%), 16 (16.5%), and 23 (23.7%) reached an MMR at weeks 12, 24, and 96, respectively. Of 18 patients on asciminib with a *BCR::ABL1* ratio >1%, at week 24, the estimated cumulative incidence of a *BCR::ABL1* ratio <1% was 22.2% by 1 year and 38.9% by 2 years. The results showed that responses continued to deepen over time with asciminib in patients previously treated with more than two lines of therapy, with additional patients achieving an MMR at later timepoints [9]. Several trials are ongoing testing this agent not only in third- but also in second- and first-line treatment. This current scenario prompts the issue of the perception of this new agent among the clinicians. Therefore, we asked about how the therapeutic strategies may change with the future prescription of asciminib in the Italian clinical practice in a GIMEMA survey assessing the number of patients currently treated in the third line in Italy, the current approach to the third line by the Italian physicians, the future role of this drug considering the reason to switch to asciminib, and the role of possible combinations or in earlier lines.

## 2. Materials and Methods

Survey data were collected and managed using the REDCap electronic data capture tools hosted at the GIMEMA Foundation (Rome, Italy) [10]. The survey invitation was sent on 31 January 2023. Data were exported on 6 April 2023. Sixty-six centers compiled the survey: 48 (73%) hospital and 18 (27%) academic centers. The median number of years of clinical practice of participants was 20 (range 1–40). In the survey, several items were collected: in particular, we collected the perceptions of academic and hospital centers, the number of total patients treated, and the number of patients in later lines switched for resistance and/or intolerance. Moreover, we collected information about the knowledge of Italian physicians about asciminib and its mechanism of action, the reasons to switch to this drug, and the potential use in the near future, suggesting several possible algorithms in patients with and without T315I mutation. We also explored the potential use of the drug in combination, in elderly patients, and in earlier lines. The original survey is included in the Supplementary Material. The survey refers to a total number of 8648 patients with a median of 80 patients followed per year by participants.

## 3. Results

**Figure 1:**
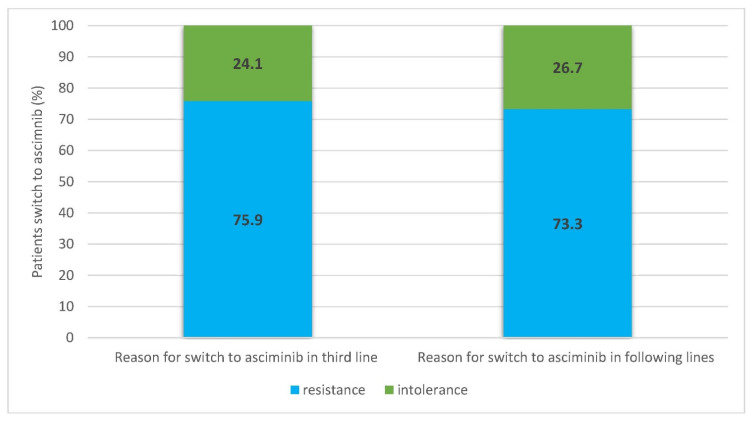
Reasons for switch to asciminib in third or following lines.

Eighty-nine percent of answering centers have familiarity with the therapeutic switch in the third line. The median number of patients treated in the third line at the time of the survey was 13 (range 0–90) for a whole cohort of 1046 patients. In the last 10 years, in an overall cohort of 1413 patients who required a third-line treatment, 60% of them had a change to a third line due to resistance, and 40% were switched due to intolerance. Overall, the centers reported that 1193 patients needed more than a third line in the last 10 years, 60% for resistance to a third line and 40% for intolerance. Before alternative treatments in later lines, 86% of answering centers declared to perform a mutational analysis with NGS as the main tool in 58% and Sanger sequencing in the remaining centers. In intolerant patients who required more than two lines of treatment, a switch to later lines was performed in 50% due to the recurrence of side effects, in 33% for the onset of cardiovascular adverse events, in 12% due to pleuropulmonary side effects, and in 5% for metabolic disorders. The preferred option used in the third line is ponatinib by 51% of the investigators, bosutinib by 35%, dasatinib by 7%, and nilotinib by 7%. Considering the possible future role of asciminib and the current use of this drug in investigational trials or as compassionate use, we asked some questions about the drug. Ninety-seven percent of clinicians knew about its mechanism of action, but only sixty-two percent had used the drug (61% as compassionate use) at the time of the survey. Overall, in the centers involved in the survey, 87 patients were treated in the third line with the drug: 76% received asciminib for resistance to a second line and 24% for intolerance (Figure 1). In 60 patients, the drug was used in the fourth or following line of treatment, with 73% of them for resistance and 27% for intolerance to previous treatments (Figure 1).

**Figure 2:**
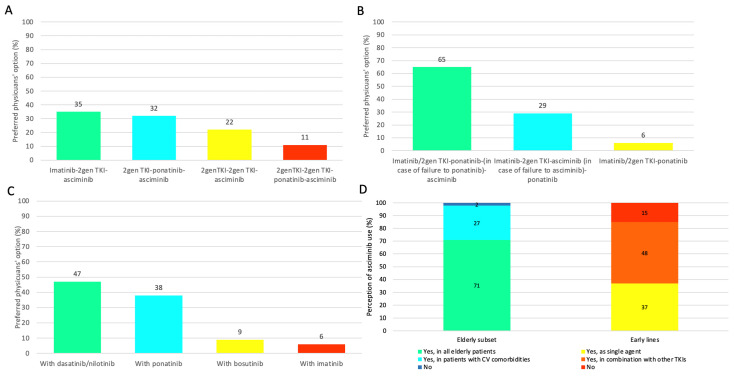
Perception of Italian physicians about the role of asciminib in the future management of CML patients: (**A**) Preferred option in resistant patients without T315I. (**B**) Best approach in case of T315I mutation. (**C**) Best combination for resistant patients. (**D**) Possible future use of asciminib in the early lines and elderly patients.

Finally, we asked about the perceptions of clinicians about the future use of the drug, considering some specific scenarios. In a resistant patient without the T315I mutation, 35% of physicians will opt for third-line asciminib after first-line imatinib and a second-line 2gen TKI without ponatinib treatment while 32% will opt for third-line asciminib if a 2gen TKI was used as a first line and rescued with second-line ponatinib (Figure 2A). Twenty-two percent of physicians will use asciminib after 2gen TKI first and second lines, and only eleven percent will use it in the fourth line after 2gen TKI first and second lines and third-line ponatinib (Figure 2A). In the case of the T315I mutation, the best approach in the near future for 65% of physicians will be the use of asciminib in the third line after ponatinib treatment and for only 29% before ponatinib (Figure 2B). Ninety-seven percent of participants believe that there is room for possible combination treatment with asciminib in the future for resistant patients and that the best combination is with 2gen TKIs (dasatinib/nilotinib) (47%) or with ponatinib (38%). Only 9% believe that the best combination is with bosutinib and 6% with imatinib (Figure 2C). Indeed, 63% of participants believe that combination treatment with asciminib could be a valid option even for intolerant patients. We also analyzed the results of suggested questions according to the different perceptions between academic and hospital centers. In resistant patients without the T315I mutation, similar results were obtained in academic and hospital centers, with third-line asciminib after first-line imatinib and a second-line 2gen TKI without ponatinib treatment in 33% and 36%, respectively. Third-line asciminib after a first-line 2gen TKI and rescued with second-line ponatinib was indicated by 33% and 32% of centers, respectively. In the case of the T315I mutation, 50% of academic-center-based and 71% of hospital-based physicians will use asciminib after ponatinib failure, but more academic centers (39%) will use asciminib before ponatinib compared to hospital-based (24%). Regarding the future combinations of asciminib with other TKIs, the differentiated results between academic and hospital centers confirmed the overall results, except for the possible association with bosutinib that was reported only by the hospital-based physicians. Seventy-one percent of participants believe that asciminib could be a valid therapeutic strategy for all elderly patients, whereas twenty-seven percent believe it could be valid only for older patients affected by comorbidities (Figure 2D). Analyzing the results between academic and hospital centers, the majority of academic physicians will use asciminib in all elderly patients (83% vs. 67%), whereas more hospital-based physicians will use asciminib only in elderly patients with severe comorbidities (31% vs. 17%). We asked about the possible use of this agent in early lines of treatment: 37% of participants believe in its role as a single agent in newly diagnosed patients, 48% believe it could be used as combination treatment, and 15% of physicians are not convinced that the drug could change the results achieved with imatinib or with 2gen TKIs (Figure 2D). In this question, no particular differences were revealed between academic and hospital-based physicians. Regarding the management of the drug, 59% of physicians believe that cardiovascular monitoring should be performed before and during treatment because data about cardiovascular toxicity are still limited, without specific differences between academic and hospital-based physicians.

## 4. Discussion

Limited options exist for CML patients who experience resistance and/or severe or persistent intolerance to multiple lines of treatment [11]. Published data showed the efficacy of a third-line treatment with bosutinib: the BYOND study reported that the median dose intensity was 300 mg/day and that more than 70% of patients must reduce the dose due to specific side effects such as increased transaminases and gastrointestinal events. The maximum benefit in this trial was observed for patients who switched to bosutinib as a second line after imatinib failure, whereas a limited rate of responses was reported for patients treated in the third or fourth line of therapy after multiple failures [12]. Another option is ponatinib; the recent OPTIC trial randomized resistant patients in the third line or over three different doses of the drug, exploring the optimization to a low dose (15 mg/day) after the achievement of the primary endpoint (*BCR::ABL1* ratio <1% at 12 months). The last follow-up showed that the maximum benefit was reached with 45 mg/day as the starting dose for resistant patients (T315I or other mutations, loss of complete hematologic response at baseline). Despite the de-escalation performed in this trial, 12% of patients treated with a higher dose of ponatinib experienced arterial occlusive events (6% as grade 3/4), suggesting that a selection of patients should be considered before switching to a third-generation TKI [13]. Among the other novel strategies, asciminib may be the most promising, considering its completely different mechanism of action. The recent follow-up at 4 years of the phase I trial that enrolled heavily pre-treated patients with more than three previous TKIs in more than 70% of cases, showed, in the non-T315I mutated cohort, a cumulative incidence of the MMR in patients who entered without this response at a baseline of 58%. The advantage was similar regardless of the previous lines of therapies received and for patients with a molecular burden <10%. In patients with a *BCR::ABL1* ratio >10%, 32% of them achieved at least an MMR after 4 years [7]. A managed access program (MAP) was supported all over the world for patients in the third or later lines of treatment: a recent update of the Italian MAP on 77 patients enrolled between April 2019 and October 2022 was reported. After a median follow-up of 8.5 months, 54.6% of patients improved the baseline response with similar efficacy within resistant and intolerant patients. Efficacy was reported even in T315I-mutated patients treated with an increased dose of 200 mg BID with none of the patients worsening the baseline response and 54.6% of them improving the response. The final analysis showed that an advantage was evident in ponatinib naïve vs. pre-treated patients, with at the last response 24 naïve patients (70.6%) achieving an improvement compared to 18 pre-treated patients (41.9%). In 64 patients who entered the program with less than an MMR, 19 out of 30 ponatinib naïve patients achieved an MMR (63.3%) compared to 12 out of 34 ponatinib pre-treated patients (35.3%) [14]. In Italy, a 97.6% increase in third-line treatments in CML patients has been demonstrated in an analyzed period between 2015 and 2018 [15]. The present survey results showed that most patients required an alternative treatment for resistance, but only 86% of centers performed a mutational analysis before switching to using NGS in 58% of the cases. Ponatinib is the best option suggested in the third line even if half of physicians answered that they would switch later lines with alternative second-generation TKIs. The majority of physicians interviewed have knowledge of asciminib’s mechanism of action but have limited clinical experience, mostly in compassionate use with few cases, mainly after resistance to multiple lines of therapies. In the case of resistance not due to the T315I mutation, most of the physicians will adopt asciminib as the third line both in the case of patients who started with first-line imatinib and experienced failure with a second-line 2gen TKI and in the case of patients who started with a first-line 2gen TKI and experienced failure with second-line ponatinib. Indeed, in the case of resistance due to the T315I mutation, most physicians are still convinced about the early use of ponatinib and to rescue patients with asciminib only as the third line. This result is probably influenced by few data shown in this setting and by the missing indication by the EMA which did not accept, as did the FDA, the drug for T315I-mutated patients. Due to its peculiar new mechanism of action, asciminib was created for possible combinations, and most clinicians believe that these strategies could be feasible even in intolerant patients. In real-world Italian data, more than half of the patients were elderly with a high-comorbidity profile. It is a common perception that the drug could be a valid option in this setting, even if more than half of physicians are convinced that more safety data are warranted to exclude potential long-term cardiovascular toxicity, even if not demonstrated in the recent 4-year median follow-up of a phase 1 trial [7]. Investigational trials testing the drug in the first line are still ongoing, and results are not yet described: for this reason, only a small fraction of the participants of the survey believe that the drug could replace in the long-term the available TKIs and improve the results obtained. In conclusion, asciminib could be a valid strategy for CML patients in the third or later lines as shown by sponsored trials and results in real-world evidence. The results of this survey showed that clinicians are aware of the potentiality of this agent and have already explored its possible use in different settings, including moving it forward in the future and anticipating it considering the improved safety profile.

## Data Availability

Not applicable.

## Supplementary Materials

The following supporting information can be downloaded at https://www.mdpi.com/article/10.3390/jcm12165267/s1, Original GIMEMA survey.

## Appendix A

**Table:**

AOU OSPEDALI RIUNITI “UMBERTO I—G.M. LANCISI—G. SALESI”—ANCONA—SOD CLINICA EMATOLOGICA, Serena Rupoli
IRCCS CENTRO DI RIFERIMENTO ONCOLOGICO DI AVIANO—SOSD ONCOEMATOLOGIA TRAPIANTI EMOPOIETICI E TERAPIE CELLULARI, Maria Grazia Michieli
AOU CONSORZIALE POLICLINICO—BARI—UO EMATOLOGIA CON TRAPIANTO, Pellegrino Musto
ASST PAPA GIOVANNI XXIII—OSPEDALE DI BERGAMO—SC EMATOLOGIA, Alessandro Rambaldi
AOU DI BOLOGNA—POLICLINICO S. ORSOLA-MALPIGHI—UOC EMATOLOGIA, Fausto Castagnetti
AO BROTZU, PRESIDIO OSPEDALIERO A. BUSINCO—CAGLIARI—SC EMATOLOGIA E CTMO, Giovanni Caocci
CTC U.O DI EMATOLOGIA CON TRAPIANTO DI MIDOLLO OSSEO—CATANIA, Francesco Di Raimondo
AO DI CATANZARO “PUGLIESE-CIACCIO”, PRESIDIO OSPEDALIERO “CIACCIO—DE LELLIS”—EMATOLOGIA, Luciano Levato
AOU CAREGGI—FIRENZE—SOD EMATOLOGIA, Antonella Gozzini
AO DI RILIEVO NAZIONALE ANTONIO CARDARELLI—NAPOLI—UOC EMATOLOGIA CON TRAPIANTO DI MIDOLLO, Mario Annunziata
AOU FEDERICO II—NAPOLI—UOC EMATOLOGIA, Fabrizio Pane
AOU POLICLINICO P. GIACCONE—PALERMO—UO EMATOLOGIA, Sergio Siragusa
AO OSPEDALI RIUNITI VILLA SOFIA CERVELLO—PALERMO—UO EMATOLOGIA AD INDIRIZZO ONCOLOGICO, Caterina Patti
AO DI PERUGIA, OSPEDALE S. MARIA DELLA MISERICORDIA—EMATOLOGIA E TRAPIANTO MIDOLLO OSSEO, Cristina Mecucci
AO OSPEDALI RIUNITI MARCHE NORD—OSPEDALE SAN SALVATORE—PESARO—UOC EMATOLOGIA E CENTRO TRAPIANTI, Giuseppe Visani
027, FONDAZIONE POLICLINICO UNIVERSITARIO AGOSTINO GEMELLI IRCCS—ROMA—AREA EMATOLOGICA, Simona Sica
UNIVERSITA’ DEGLI STUDI DI ROMA “SAPIENZA”—DIPARTIMENTO DI MEDICINA TRASLAZIONALE E DI PRECISIONE—U.O.C. EMATOLOGIA, Massimo Breccia
ASL ROMA 2, OSPEDALE S. EUGENIO—OSPEDALE S.EUGENIO—UOC EMATOLOGIA, Elisabetta Abruzzese
AOU DI SASSARI—CLINICHE UNIVERSITARIE—STABILIMENTO CLINICHE DI SAN PIETRO —UOC EMATOLOGIA, Claudio Fozza
ASUI DI UDINE—PRESIDIO OSPEDALIERO “SANTA MARIA DELLA MISERICORDIA”—CLINICA EMATOLOGICA, Mario Tiribelli
AULSS 8 BERICA—OSPEDALE DI VICENZA—UOC EMATOLOGIA, Maria Cristina Miggiano
IRCCS AOU SAN MARTINO—GENOVA—UO EMATOLOGIA E TRAPIANTI, Germana Beltrami
AOU CITTA’ DELLA SALUTE E DELLA SCIENZA, OSPEDALE S. GIOVANNI BATTISTA MOLINETTE—TORINO—SC EMATOLOGIA 2, Patrizia Pregno
AOU DI PARMA—SC EMATOLOGIA E CENTRO TRAPIANTI MIDOLLO OSSEO, Monica Crugnola
FONDAZIONE IRCSS POLICLINICO SAN MATTEO—PAVIA—UO EMATOLOGIA, Luca Arcaini
AOU INTEGRATA DI VERONA, POLICLINICO G.B. ROSSI—UOC EMATOLOGIA, Massimiliano Bonifacio
FONDAZIONE IRCCS CA’ GRANDA, OSPEDALE MAGGIORE POLICLINICO—MILANO—EMATOLOGIA—PADIGLIONE MARCORA, Alessandra Iurlo
ASO S. CROCE E CARLE—CUNEO—SC EMATOLOGIA, Davide Rapezzi
IRCCS OSPEDALE S. RAFFAELE—MILANO—UO ONCOEMATOLOGIA, Fabio Ciceri
ASST DEGLI SPEDALI CIVILI DI BRESCIA—UO EMATOLOGIA, Alessandra Tucci
ASL NAPOLI 1 CENTRO, PRESIDIO OSPEDALIERO ASCALESI—OSPEDALE S.MARIA DI LORETO NUOVO, Angiolina Rocino
AUSL DI REGGIO EMILIA —ARCISPEDALE SANTA MARIA NUOVA, IRCCS—SC EMATOLOGIA, Francesco Merli
ASL BRINDISI, OSPEDALE ‘PERRINO’—BRINDISI—UO EMATOLOGIA, Domenico Pastore
AOU ARCISPEDALE SANT’ANNA—CONA (FE)—UOC EMATOLOGIA E FISIOPATOLOGIA DELLA COAGULAZIONE, Francesco Cavazzini
AOU PISANA—UO EMATOLOGIA UNIVERSITARIA, Sara Galimberti
AOU SENESE—UOC EMATOLOGIA E TRAPIANTI, Monica Bocchia
AULSS 3 SERENISSIMA, OSPEDALE DELL’ANGELO—MESTRE—UO EMATOLOGIA, Renato Bassan
UOSD di Ematologia Presidi Osp. S.Spirito-Nuovo Regina Margherita—S.Filippo Neri, Tommaso Caravita di Toritto
APSS TRENTINO, OSPEDALE DI TRENTO—PRESIDIO OSPEDALIERO S.CHIARA—SSD EMATOLOGIA, Anna Guella
ASST FATEBENEFRATELLI SACCO OSPEDALE LUIGI SACCO—POLO UNIVERSITARIO —EMATOLOGIA E MEDICINA TRASFUSIONALE (SIMT), MARIA CRISTINA CARRARO
AOU DI PADOVA—UO EMATOLOGIA, Gianni Binotto
CASA DI CURA LA MADDALENA S.P.A.—DIPARTIMENTO ONCOLOGICO DI III LIVELLO—PALERMO—UO ONCOEMATOLOGIA E TMO, Maurizio Musso
ISTITUTI FISIOTERAPICI OSPITALIERI—IFO—ISTITUTO REGINA ELENA—ROMA—UOSD EMATOLOGIA, Andrea Mengarelli
ASP MESSINA, PRESIDIO OSPEDALIERO ‘S. VINCENZO’—TAORMINA—UOC EMATOLOGIA E LABORATORIO DI BIOLOGIA MOLECOLARE, Giuseppe Mineo
AREA VASTA N. 5 ASCOLI PICENO—S. BENEDETTO DEL TRONTO, PRESIDIO OSPEDALIERO AV5 OSP. GEN. PROV.LE “C.G.MAZZONI” —UOC EMATOLOGIA, Piero Galieni
AOU POLICLINICO “G. MARTINO”—MESSINA—UOC EMATOLOGIA, Caterina Musolino
AOU MAGGIORE DELLA CARITA’ DI NOVARA—SCDU EMATOLOGIA, Monia Lunghi
AOU SANT`ANDREA—ROMA—UOC EMATOLOGIA, Agostino Tafuri
AZIENDA SANITARIA UNIVERSITARIA INTEGRATA DI TRIESTE—SC EMATOLOGIA, Francesco Zaja
ASL LECCE, OSPEDALE ‘V. FAZZI’—UO EMATOLOGIA, Nicola Di Renzo
ASST DEGLI SPEDALI CIVILI DI BRESCIA—SSVD CENTRO TRAPIANTI MIDOLLO PER ADULTI—CATTEDRA DI EMATOLOGIA, Domenico Russo
ASL DELLA PROVINCIA DI BARLETTA, ANDRIA, TRANI, OSPEDALE “MONS. DIMICCOLI”—BARLETTA—UO EMATOLOGIA, Giuseppe Tarantini
I.R.S.T. SRL IRCCS—MELDOLA—SC ONCOLOGIA MEDICA, Alessandro Lucchesi
U.O.C. ONCOEMATOLOGIA “ISTITUTO ONCOLOGICO VENETO IRCCS, PRESIDIO OSPEDALIERO S. GIACOMO APOSTOLO, Roberto Sartori
ASL LATINA, PRESIDIO OSPEDALIERO NORD—OSPEDALE SANTA MARIA GORETTI—UOC EMATOLOGIA, Alessandro Pulsoni
ARNAS GARIBALDI, PO GARIBALDI-NESIMA—CATANIA—UOC EMATOLOGIA, Ugo Consoli
IRCCS ONCOLOGICO ISTITUTO TUMORI GIOVANNI PAOLO II—BARI —UO EMATOLOGIA, Attilio Guarini
ASP DI RAGUSA—UO IMMUNOEMATOLOGIA E MEDICINA TRASFUSIONALE, Sergio Cabibbo
ASP CALTANISSETTA—UO EMATOLOGIA, Maria Flavia Fiorenza
OSPEDALE MAURIZIANO UMBERTO I—TORINO—SCDU EMATOLOGIA, Carmen Fava
OSPEDALE DI SASSUOLO SPA—EMATOLOGIA, Sara Bigliardi
ASL TO 2, TORINO NORD EMERGENZA SAN GIOVANNI BOSCO—SSD EMATOLOGIA, Massimo Pini
OSPEDALE S.ANDREA DI VERCELLI—ASL VERCELLI—SS EMATOLOGIA, LORENZO DE PAOLI
ASL DI SALERNO— PO SAN LUCA, Giovanni D’Arena
ASST-MANTOVA-Struttura Complessa-Servizio immunoematologia e Medicina Trasfusionale-Ospedale San Carlo Poma, Massimo Franchini
LSS n.7-PEDEMONTANA- OSPEDALE DI BASSANO DEL GRAPPA UOC ONCOEMATOLOGIA, Eros Di Bona

## References

[1] Bower H., Björkholm M., Dickman P.W., Höglund M., Lambert P.C., Andersson T.M.L. (2016). Life expectancy of patients with chronic myeloid leukemia approaches the life expectancy of the general population. J. Clin. Oncol..

[2] Breccia M., Olimpieri P.P., Olimpieri O., Pane F., Iurlo A., Foggi P., Cirilli A., Colatrella A., Cuomo M., Gozzo L. (2020). How many chronic myeloid leukemia patients who started a frontline second-generation tyrosine kinase inhibitor have to switch to a second-line treatment? A retrospective analysis from the monitoring registries of the Italian medicines agency (AIFA). Cancer Med..

[3] Cortes J., Lang F. (2021). Third-line therapy for chronic myeloid leukemia: Current status and future directions. J. Hematol. Oncol..

[4] Steegmann J.L., Baccarani M., Breccia M., Casado L.F., García-Gutiérrez V., Hochhaus A., Kim D.-W., Kim T.D., Khoury H.J., Le Coutre P. (2016). European LeukemiaNet recommendations for the management and avoidance of adverse events of treatment in chronic myeloid leukemia. Leukemia.

[5] Senapati J., Sasaki K., Issa G.C., Lipton J.H., Radich J.P., Jabbour E., Kantarjian H.M. (2023). Management of chronic myeloid leukemia in 2023-common ground and common sense. Blood Cancer J..

[6] Shanmuganathan N., Hughes T.P. (2022). Asciminib for chronic myeloid leukaemia: Next questions. Br. J. Haematol..

[7] Mauro M., Hughes T.P., Kim D.W., Rea D., Cortes J.E., Hochhaus A., Sasaki K., Breccia M., Talpaz M., Ottmann O. (2023). Asciminib monotherapy in patients with CML-CP without BCR::ABL1 T315I mutations treated with at least two prior TKIs: 4-year phase 1 safety and efficacy results. Leukemia.

[8] Hochhaus A., Réa D., Boquimpani C., Minami Y., Cortes J.E., Hughes T.P., Apperley J.F., Lomaia E., Voloshin S., Turkina A. (2023). Asciminib vs. bosutinib in chronic-phase chronic myeloid leukemia previously treated with at least two tyrosine kinase inhibitors: Longer-term follow-up of ASCEMBL. Leukemia.

[9] Hughes T., Rea D., Boquimpani C., Minami Y., Mauro M., Cortes J.E., Apperley J.F., Gutiérrez V.G., Kapoor S., Allepuz A. (2022). Dynamics of response and response factors in patients with chronic myeloid leukemia in chronic phase (CML-CP) after >2 prior tyrosine kinase inhibitors (TKIs) in the phase 3 Ascembl study. Blood.

[10] Harris P.A., Taylor R., Minor B.L., Elliott V., Fernandez M., O’Neal L., McLeod L., Delacqua G., Delacqua F., Kirby J. (2019). The REDCap consortium: Building an international community of software platform partners. J. Biomed. Inform..

[11] García-Gutiérrez V., Breccia M., Jabbour E., Mauro M., Cortes J.E. (2022). A clinician perspective on the treatment of chronic myeloid leukemia in the chronic phase. J. Hematol. Oncol..

[12] Hochhaus A., Gambacorti-Passerini C., Abboud C., Gjertsen B.T., Brümmendorf T.H., Smith B.D., Ernst T., Giraldo-Castellano P., Olsson-Strömberg U., Saussele S. (2020). Bosutinib for pretreated patients with chronic phase chronic myeloid leukemia: Primary results of the phase 4 BYOND study. Leukemia.

[13] Cortes J., Apperley J., Lomaia E., Moiraghi B., Undurraga Sutton M., Pavlovsky C., Chuah C., Sacha T., Lipton J.H., Schiffer C.A. (2021). Ponatinib dose-ranging study in chronic phase chronic myeloid leukemia: A randomized, open-label phase 2 clinical trial. Blood.

[14] Breccia M., Russo Rossi A., Giai V., Martino B., Fava C., Annunziata M., Abruzzese E., Binotto G., Baratè C., Nardozza A.P. (2023). Real-World Efficacy Profile of Asciminib in An Italian, Multi-Resistant Chronic Phase Chronic Myeloid Leukemia (CML-CP) Patient Population. HemaSphere.

[15] Breccia M., Chiodi F., Nardozza A.P., Valsecchi D., Perrone V., Sangiorgi D., Giacomini E., Rendace M.C., Coco P., Premoli E. (2022). Real-world analysis of therapeutic management and disease burden in chronic myeloid leukemia patients with later lines in Italy. J. Clin. Med..

