# Supplementary material for: Italian Physicians’ Perceptions about the Role of Asciminib in Later Lines Chronic Myeloid Leukemia in Clinical Practice: A GIMEMA Survey
Source: J Clin Med. 2023 Aug 13;12(16):5267. doi: 10.3390/jcm12165267 (PMC10455524; doi:10.3390/jcm12165267)
Supplement: Supplementary file 1 [file jcm-2534205-supplementary.pdf › jcm-2534205-supplementary.pdf]

Survey GIMEMA on the role of Asciminib in the treatment of CML patients

Dear Prof/Dr,

Tyrosine kinase inhibitors (TKIs) ensure to the majority of CML patients a life expectancy superimposable to the normal population. However, a significant proportion of patients experience failure to treatment with first- or second-generation TKIs.

Asciminib, the first example of allosteric inhibitor, has been recently approved by FDA and EMA as third line treatment in chronic phase chronic myeloid leukemia (CP-CML) patients after resistance and/or intolerance to almost two previous lines of treatment. Indeed, in the phase 3 ASCEMBL study comparing asciminib and bosutinib, MMR rate was superior in the asciminib arm together with a favorable safety profile.

Although these promising results, it is not clear how the introduction of this new TKI will change the therapeutic scenario for CML patients in later lines of treatment.

In the present project, we aim to analyze the perception of Italian physicians about the role of asciminib in the future management of Italian CML patients in later lines of treatment. To this end, we will investigate:

Approximate number of patients currently treated in 3L in ItalyApproach to 3L of Italian physicians (i.e. preferred TKI, reason of switch)Role of asciminib in 3L (i.e. rate of patients switched to asciminib, reason of switch to asciminib, possible combinations, role in early lines)We invite you to fill the present survey.

Please note that the survey can be filled in multiple sessions by selecting the "Save and Return later" option.

For any request of assistance, please contact survey@gimema.it

Kind regards,

Massimo Breccia

Where do you practice your clinical activity?	◯ Academic ◯ Hospital
How many years of clinical practice for CML treatment?	______________________
Number of CML patients in your center	______________________
How many CML patients do you follow per year?	______________________

Treatment of CML patients in third or following lines

Have you ever treated patients in third line?	◯ Yes ◯ No
How many patients are actually treated in third line in your center?	______________________
How many patients switched to third line for resistance in your center? (in the last 10 years)	______________________

How many patients switched to following lines for resistance in your center? (in the last 10 years)	______________________
Did you test the mutation profile before switch for resistance?	◯ Yes ◯ No
Which is the tool that was used for mutations in your center	◯ Sanger ◯ NGS
How many patients switched to third line for intolerance in your center? (in the last 10 years)	______________________
How many patients switched to following lines for intolerance in your center? (in the last 10 years)	______________________
Which was the main reason that induce you to switch for intolerance?	◯ Recurrence of side effect<br>◯ Cardiovascular AEs<br>◯ Pulmonary AEs<br>◯ Metabolic AEs
Which was the preferred option that you used as third line? (report the % of each option)	☐ Nilotinib ______<br>☐ Dasatinib ______<br>☐ Bosutinib ______<br>☐ Ponatinib ______

Asciminib

Do you know the mechanism of action of the new TKI, asciminib?	◯ Yes ◯ No
Have you used asciminib in your center?	◯ Yes ◯ No
Did you use asciminib as compassionate use?	◯ Yes ◯ No
How many patients with resistance did you switch to asciminib in third line?	______________________
How many patients with resistance did you switch to asciminib in following lines?	______________________
How many patients with intolerance did you switch to asciminib in third line?	______________________
How many patients with intolerance did you switch to asciminib in following lines?	______________________
What do you think is the best way to use asciminib in a resistant patients without T315I mutation?	◯ Imatinib-2gen TKI-asciminib<br>◯ 2genTKI-2gen TKI-asciminib<br>◯ 2genTKI-2gen TKI-ponatinib-asciminib<br>◯ 2gen TKI-ponatinib-asciminib

In case of T315I mutation, what do you think would be the best approach in the future?	◯ Imatinib/2gen TKI-ponatinib<br>◯ Imatinib/2gen TKI-ponatinib-(in case of failure to ponatinib)-asciminib<br>◯ Imatinib-2gen TKI-asciminib (in case of failure to asciminib)-ponatinib
Asciminib works with a different mechanism of action and could be used in combination: do you think there is room in the CML therapeutic scenario for a combination strategy in resistant patients?	◯ Yes ◯ No
In your opinion, which will be the best combination for resistant patients?	◯ With imatinib<br>◯ With dasatinib/nilotinib<br>◯ With ponatinib<br>◯ With bosutinib
Asciminib works with a different mechanism of action and could be used in combination: do you think there is room in the CML therapeutic scenario for a combination strategy in intolerant patients?	◯ Yes ◯ No
Do you think asciminib could be used in elderly subset?	◯ Yes, in all elderly patients<br>◯ Yes, in patients with CV comorbidities<br>◯ No
Do you think asciminib could play a role in the early lines?	◯ Yes, as single agent<br>◯ Yes, in combination with other TKIs<br>◯ No
In your opinion, a cardiovascular monitoring should be performed before and during asciminib?	◯ No, because no CV adverse events were reported<br>◯ Yes, always<br>◯ Yes, because data about CV toxicity are limited
